# Comparison of two non-invasive arterial blood pressure monitoring techniques in brown bears (*Ursus arctos*)

**DOI:** 10.1016/j.vas.2020.100094

**Published:** 2020-01-20

**Authors:** Jacopo Morelli, Angela Briganti, Boris Fuchs, Ðuro Huber, Alina L. Evans, Slaven Reljić, Jon M. Arnemo

**Affiliations:** a Department of Forestry and Wildlife Management, Inland Norway University of Applied Sciences, Anne Evenstadvegen 80, 2480 Koppang, Norway; b Department of Veterinary Sciences University of Pisa, Via Livornese, 56122 San Piero A Grado, Italy; c Department of Biology, Faculty of Veterinary Medicine, University of Zagreb, Heinzelova ul. 55, 10000 Zagreb, Croatia; d Department of Wildlife, Fish and Environmental Studies, Swedish University of Agricultural Sciences, Skogsmarksgränd 17, 901 83 Umeå, Sweden

**Keywords:** Brown bear, Blood pressure, Korotkoff, Oscillometry, Hypertension, Sphygmomanometer, BP, blood pressure, HR, heart rate, XK, xylazine-ketamine, IM, intramuscularly, GPS, Global Positioning System, MZT, medetomidine-zolazepam-tiletamine, VHF, Very High Frequency, SpO_2_, hemoglobin-oxygen saturation, SAP, systolic arterial blood pressure, DAP, diastolic arterial blood pressure, MAP, mean arterial blood pressure, CW/LC, cuff width/limb circumference, SD, Standard Deviation, LoA, limit of agreement

## Abstract

Monitoring arterial blood pressure (BP), represents a more accurate evaluation of hemodynamics than heart rate alone and is essential for preventing and treating intra- and post-operative complications in wildlife chemical immobilization.

The objectives of the study were to test the correlation between standard oscillometry and Korotkoff's technique in anesthetized free-ranging brown bears in Croatia and Scandinavia and to assess the blood pressure in both locations.

Five bears were snared and darted with xylazine and ketamine in Croatia, and 20 bears were darted from a helicopter with medetomidine and tiletamine-zolazepam in Scandinavia. Blood pressure was simultaneously measured with both techniques every 5 minutes. Correlation between techniques, trends of BP variation, and the factors of the capture which likely influenced BP were assessed.

Successful measurements of BP were achieved in 93% of all attempts with the Korotkoff's technique but in only 29% of all attempts with oscillometry. The latter method mostly provided lower values of BP compared to Korotkoff's technique in yearlings. Most bears showed a decreasing trend in systolic and mean BP over time, consistent between the two techniques. All bears were hypertensive: the auscultatory technique detected moderate to severe systolic hypertension in 25% and 84% of bears in Croatia and in Scandinavia, respectively, with significantly higher BP in subadults and adults compared to yearlings. Only Korotkoff's method resulted in a reliable and effective tool for BP assessment in brown bears. The anesthetic protocols used in the present study in association with the capture methods produced hypertension in all animals.

## Introduction

Arterial blood pressure (BP), which is strictly connected to heart rate (HR), stroke volume and systemic vascular resistance, provides a better overview of the cardiovascular status of anesthetized patients, compared to HR alone. Hence, monitoring BP improves the outcome of anesthesia by helping to prevent, diagnose early and to treat a wide variety of complications. Previous studies assessed the efficacy of multiparametric oscillometric devices on captive and wild brown bears (Nelson et al., 2003; Teisberg et al., 2014) and other bear species (Jeong et al., 2017), with controversial results. Some studies resulted in an overall good agreement between invasive and non-invasive techniques whilts significant differences emerged from others. Moreover, to date, no studies assessed the precision, accuracy and reliability of BP monitoring in anesthetized brown bears by using the Korotkoff's technique, which is routinely performed in hospital settings in human medicine (Canzanello et al., 2001; Carlson et al., 2019). This technique, when performed by an expert listener, is considered to be the most accurate non-invasive method, and is accepted as a reference standard for assessing the accuracy of other non-invasive BP methods (Nitzan et al., 2013).

Assessing a quick, easy, reliable and accurate method to measure BP would further enhance monitoring and prevention of cardiovascular complications in anesthetized brown bears in field procedures.

The objectives of the present study were to assess how the different immobilization method affected the BP of anesthetized free-ranging brown bears, measured by two different non-invasive techniques (standard oscillometry and Korotkoff's or auscultatory technique), both in Croatia and Scandinavia, and to test reliability and correlation of both techniques. The present study also aimed to verify whether hypertension occurred in both capture contexts or not, to estimate its stage, to describe BP trends throughout the procedures, and finally to find out what are the factors which likely influenced BP the most.

No similar studies on non-invasive BP have been developed on wild free-ranging brown bears within these two specific immobilization contexts.

## Materials and methods

### Study area and animals

The study included five chemical immobilizations of free-ranging brown bears in Plitvice Lakes National Park, Croatia (44.8° N, 15.5° E), 300–800 m above sea level, as part of an ongoing research project. Three adult bears (one female and two males, one male was captured twice), and one male yearling (1-year-old) were trapped in April and May 2016, during the night and early morning (between 7 p.m. and 6 a.m.) with ambient temperatures (*T_a_*) ranging from 5 °C to 16 °C.

In the second part of the study, 20 free-ranging brown bears were chemically immobilized in the counties of Dalarna, Sweden (61° N, 15°E) and Hedmark, Norway (60° N, 11° E), 300–700 m above sea level, within the Scandinavian Brown Bear Research Project's ongoing research projects. The captures took place in April and May 2017, during the day (9 a.m. to 7 p.m.). *T_a_* ranged from 4°C to 22°C. The Scandinavian bears were divided into two groups based on age: Group A consisted of yearlings (*n* = 7), whereas group B consisted of subadults (Græsli et al., 2015) (2–4 years old, *n* = 2) and adults (*n* = 11).

### Capture methods, drugs and darting materials

In Croatia, bears were trapped with spring-activated foot snares equipped with a GSM-based alarm. The duration of the total exertion of the bears in the foot snare was considered from the reception of the trap alarm to the recumbency time due to the effect of the anesthetic drugs.

The bears were darted with a combination of xylazine (Rompun®, 500 mg dry substance) and ketamine (Ketaminol® 10, 100 mg/ml) (XK). The drugs were mixed by adding 5 ml of ketamine to xylazine dry substance so that 1 ml of the solution contained 90.9 mg xylazine and 90.9 mg of ketamine and administered with a CO_2_ powered rifle (Dan-Inject®, Børkop DK-7080, Denmark). Additional XK was administered after 15 min if the bear was not immobilized. Before the start of the procedures, 3–5 mg/kg of ketamine was administered intramuscularly (IM) by hand syringe depending on the anesthetic plane. Drug doses are given in Table 1. Bears were equipped with global positioning system (GPS) radio-collar (Vectronic Aerospace®, Berlin D-12489, Germany) and were left to recover close to the trap site. All bears recovered completely (confirmed by camera-traps and GPS positioning).

**Table 1:** Mean, Standard Deviation (±SD) and range values of exertion and induction time, xylazine dose (*X*), ketamine dose (*K*), medetomidine dose (*M*), zolazepam-tiletamine dose (ZT) during the chemical immobilizations in Croatia and within group A (yearlings) and group B (subadults and adults) in Scandinavia.

Croatia		Exertion (min)*	Induction (min)⁎⁎	*X* (mg/kg)	*K* (mg/kg)
**Mean**		183	17	4.54	8.25
**± SD**		69.98	5.98	1.33	3.08
**Range**		61–231	10–26	2.80–6.41	4.58–8.91

Scandinavia		Exertion (min)*	Induction (min)⁎⁎	*M* (µg/kg)	ZT (mg/kg)
**A**	**Mean**	6	3	84.71	4.23
	**± SD**	1.38	0.95	10.24	0.47
	**Range**	4–8	2–4	69.17–94.86	3.47–4.63
**B**	**Mean**	15	8	75.83	3.26
	**± SD**	13.66	10.09	34.03	1.43
	**Range**	4–52	2–39	51.55–140.84	2.58–7.00

⁎ Exertion time is expressed in minutes (min) and is considered from the activation of the trap (Croatia) or from the start of the helicopter chase (Scandinavia) to the recumbency because of the induction of the anesthesia.

⁎⁎ Induction time is expressed in minutes (min) and is considered from the darting to the recumbency due to the effect of the drugs.

In Scandinavia, bears were located by radiotracking and darted from a helicopter with a CO_2_ powered rifle (Dan-Inject®, Børkop DK-7080, Denmark).

The time of intensive helicopter pursuit for the actual darting was decided not to exceed 1 min, and the duration of the total exertion of the bears was calculated from the start of the chase until recumbency. Scandinavian bears were darted with a combination of medetomidine (Domitor®, 1 mg/ml, or Zalopine®, 10 mg/ml) and zolazepam-tiletamine (Zoletil Forte® Vet, 50 mg/ml) (MZT) according to an established protocol (Arnemo & Evans, 2017). At the end of the procedures, medetomidine was antagonized with atipamezole (Antisedan®, 5 mg/ml) administered IM at five times the total dose of medetomidine and the bears were left to recover at the site of capture. During 30 days after the captures, the activity of all bears was monitored through telemetry and the successful recovery was confirmed in all cases. All Scandinavian bears were moved to dorsal recumbency and underwent abdominal surgery in order to implant or retrieve VHF transmitters or bio-loggers (Arnemo & Evans, 2017), and both subadult and adult bears were also equipped with GPS radio collars. Analgesia was provided by administering meloxicam subcutaneously at 0.4 mg/kg (Metacam®, 20 mg/ml).

### Monitoring

The rectal temperature was measured immediately with either a mercury or a digital thermometer, in Croatia and Scandinavia respectively, and then monitored over time during the immobilization. Assessment of anesthetic depth was done by evaluating palpebral reflex, jaw tone and positioning of the eyeball every 5 min. If required to maintain an adequate level of anesthesia, additional ketamine (2–3 mg/kg) was given IM. Heart rate and hemoglobin-oxygen saturation (SpO_2_) were continuously measured by pulse-oximetry, whereas the respiratory rate was monitored every 5 min by either using a stethoscope or counting the thoracic wall excursions over 30 s.

Both an automatic oscillometric aneroid sphygmomanometer (OMRON M6 Comfort IT® Automatic Blood Pressure Monitor, OMRON Healthcare UK Ltd; pressure range 0–299 mmHg, accuracy of ± 3 mmHg, pulse range 40–180 beats/min) and a standard aneroid sphygmomanometer (DM330LF, LOGIKO VISUAL®, Moretti S.p.a. Italy; pressure range 0–300 mmHg, accuracy of ± 2 mmHg), which consisted of a manometer, a cuff, a valve and a balloon, were used at the same time to monitor systolic and diastolic arterial blood pressure (SAP and DAP, respectively). Both instruments were purchased immediately before the first day of captures, then the standard aneroid sphygmomanometer was calibrated by means of a mercury manometer every six months until the last day of the BP monitoring.

The hair proximal to either the carpus or the tarsus was clipped, the limb circumference was measured, and a cuff of adequate size was placed, when available, for automatic readings (the manual sphygmomanometer included only a 14 cm-wide cuff, the standard size for an adult human forearm), and the ratio between the cuff width and the limb circumference (CW/LC) was calculated.

The manual technique detected Korotkoff's sounds of the pulse (so SAP and DAP, corresponding to phase I and phase V, respectively) through the diaphragm of a stethoscope (Littmann® Classic II S.E., 3M®, Minnesota, U.S.) placed above the medial plantar artery, distally from the cuff. Then, mean arterial blood pressure (MAP) was calculated from SAP and DAP values.

SAP and DAP were measured continuously and simultaneously with both techniques by the same operator, in order to obtain from one to three values every 5 min from each device. Thus, the mean values for SAP, DAP, and MAP were calculated within every 5-min gap.

SAP/DAP were measured as long as possible during the procedures in all the bears from single captures (*n* = 5 in Croatia, n = 7 in Scandinavia) and for 30 min per individual in case of family captures (*n* = 13). In case of suspected malfunction of the automatic sphygmomanometer, either a solution of soap and water was poured between the limb and the cuff in order to improve adherence and the reading, or the cuff was changed.

SAP/DAP values were corrected in relation to the size of the applied cuff using derived calculations from a study on different cuff sizes used in human patients (Maxwell et al., 1982). These corrective formulae consider a CW/LC of 0.41 as optimal. In this way, we overcame the bias of the different CW/LC_,_ though with the assumption of a similar correlation between CW/LC and overestimation (or underestimation) of SAP and DAP in bears. Thus, the corrective formulae applied to SAP and DAP values for a 14 cm-wide cuff are, respectively:

SAPc=SAPm+26.2−0.76×LC

DAPc=DAPm+16.9−0.49×LC

where the subscripts “c” and “m” indicate the corrected value and the measured value, respectively, and LC is the limb circumference. These coefficients have been extrapolated from the trendlines of the original coefficients (Maxwell et al., 1982).

SAP/DAP values were also corrected decreasing or increasing by 0.8 mmHg for each cm of the vertical distance between the inflated cuff and the right atrium level, whenever the cuff was placed more than 10 cm below or above, respectively. At the end of every daily capture session, both sphygmomanometers were simultaneously tested on one operator following the most recent American Heart Association (AHA) guidelines (Whelton et al., 2017), to confirm the agreement in SAP/DAP values and eventual malfunction. All tests showed a perfect agreement between techniques and no abnormalities.

After consulting previous studies on bear species (Caulkett & Cattet, 1997; Caulkett et al., 1999; Jeong et al., 2017; Nelson et al., 2003; Rivet et al., 2017; Teisberg et al., 2014), mild, moderate and severe hypertension was defined for both SAP and DAP: 170–200 mmHg, 201–250 mmHg and > 250 mmHg were considered systolic hypertension of mild, moderate and severe degree, respectively, whereas 110–130 mmHg, 131–170 mmHg and > 170 mmHg were considered diastolic hypertension of mild, moderate and severe degree, respectively.

### Statistical analysis

Microsoft Excel 2019 was used to conduct simple descriptive statistics including mean ± Standard Deviation (SD) and range of values for every monitored parameter, for each bear and within groups, including SAP, MAP (calculated), DAP, actual drug doses per kg, induction period (from darting to recumbency due to the anesthesia) and exertion period (struggle in the trap or helicopter chase). Weighted means and ranges are reported to account for uneven numbers of measurements per individual for SAP, MAP and DAP when describing the findings within groups e.g. group A (yearlings), group B (subadults and adults), Croatian sample, Scandinavian sample, etc. Kurtosis, skewness and their standard error were also calculated to confirm that the data within each group were normally distributed. R-3.5.3 software ("R version 3.5.1 "Feather Spray" Copyright (C)," 2018) was used to conduct the following statistical analyses. A Mann-Whitney *U* test was used to assess significant differences (*p* < 0.05) for SAP, MAP, and DAP between group A and group B in Scandinavia. Agreement and correlation between concurrent auscultatory and oscillometric SAP and DAP corrected values were analyzed using Bland–Altman test of Limits of Agreement (LoA) and the correlation coefficient (*r*) was calculated. Good agreement was considered *a priori* for |LoA| < 10 mmHg between paired BP values. Strong correlation and statistical significance were considered for |*r|* > 0.7 and *p* < 0.05 (Rosner, 2011), respectively. Regarding the Scandinavian sample, the same test was applied to group A and B, separately, and the differences were documented. The correlation for MAP values was not assessed because those values were calculated from SAP and DAP, and not directly measured. We interpreted concordance and agreement between the two instruments graphically by plotting paired corrected values for SAP and DAP. Each simultaneous pair of measurements was represented by a fix in the scatter plot, with oscillometric and auscultatory values as coordinates (“*y*” and “*x*”, respectively), and the identity function (*y*=*x*) representing the equality line. Further, the success rate, as means of the number of successful BP readings out of the number of total attempts, was calculated for both techniques in both Croatia and Scandinavia. Hence, statistical modelling on the Scandinavian data set was performed to assess variables influencing the MAP values. The “bam function” was used to fit generalized additive mixed models (Wood, 2011) with MAP as the response variable. The bam function allows fitting random effects and auto regression functions (AR1) to the model. Age of the bear in years (Age), body mass (kg), medetomidine dose per kg (M), duration of the helicopter chase in minutes (Chase), Surgical type (inserting bio-loggers and implants or their removal, which is more invasive) and Surgical status (whether the surgery was ongoing or not) were added as fixed effects. Due to only two males, we excluded sex as a factor. As the MAP measurements are unregularly spread over time post immobilization, we added a spline for a time in minutes into the fixed part using the default settings. To this full model, we added a random intercept and slope over time for each bear ID. The AR1 correlation parameter (*ρ*) was based on the residual autocorrelation of lag 1 and adjusted by testing *ρ* +/− 0.1 using the relationship estimation by maximum likelihood (fREML) scores. We then refitted the full model using the maximum likelihood (ML) method and performed a backward selection on the fixed parameters by removing the variable with the highest non-significant *p*-value until only significant terms were left.

## Results

The free-ranging brown bears were successfully anesthetized with a single dart dose during 23 of 25 captures in both countries and the time of recumbency occurred within 10–26 min (mean 17 min ± 5.98 SD) and within 2–39 min (mean 6 min ± 8.45 SD) after receiving the first dart in Croatia and in Scandinavia, respectively. The duration of the exertion was one of the parameters that differed the most among captures, and between the two countries, depending on the location of the team at the time of the trap trigger in Croatia, and on the time needed to dart from the helicopter in Scandinavia. Thus, exertion time ranged from 61 to 231 min (mean 183 min ± 69.98 SD) in Croatia, and from 4 to 52 min (mean 12 min ± 11.88 SD) in Scandinavia.

The monitoring time started 20–45 min after darting (lasting 30–60 min) in Croatia, and 15–160 min after darting (lasting 30–90 min) in Scandinavia, depending on the reliability of each immobilization and whether it was a family capture or not.

All yearlings in Scandinavia and all bears in Croatia were naive to capture, except for one individual in Croatia that was captured twice in the current study.

Detailed values (mean ± SD and range) of exertion time, induction time and drug doses within groups are presented in Table 1.

Analyzing the cardiovascular status in detail, heart rate was consistent among all the monitoring methods and it presented a generally decreasing trend in 3 of 5 bears and in 17 of 20 bears in Croatia and in Scandinavia, respectively. Yearlings presented significantly higher HR than subadults and adults. Regarding BP monitoring, all measurements with Korotkoff's method were successful, leading to repeatable values, which resulted in predictable trends of variation, with no unexpectedly different “out-of-trend” values. On an average of 172 attempts, 144 of them provided a clear auscultation of Korotkoff's pulse tones (success rate = 83.7%) in Croatia, and on an average of 1494 attempts, 1410 provided a correct measurement (success rate = 94.4%) in Scandinavia.

The automated oscillometric device provided a reading in 128 out of 384 attempts (success rate = 33.3%) in Croatia and in 630 out of 2205 attempts (success rate = 28.6%), in Scandinavia. Inconstant values occurred often in either study area.

The CW/LC ranged from 0.43 to 0.74 in Croatia, and from 0.41 to 0.87 in Scandinavia, with higher values (and higher underestimation of SAP/DAP) in yearlings. Thereby, SAP and DAP were corrected adding from 1.8 mmHg to 11.8 mmHg, and from 1.1 mmHg to 14.0 mmHg to the measured values in Croatia and Scandinavia, respectively. One yearling was excluded from the Mann–Whitney *U* test and the BP alteration analysis because of excessively high CW/LC and lack of blood pressure measurements during the anesthesia.

**Fig. 1:**
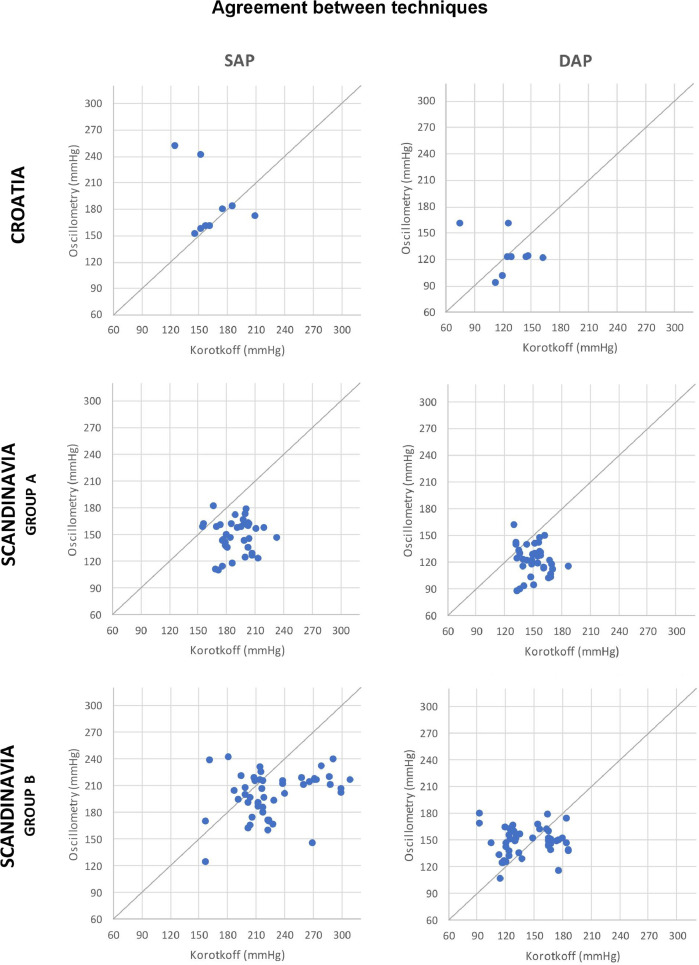
Distribution of paired corrected values for SAP and DAP simultaneously recorded by oscillometric (mmHg, *Y*-axis) and auscultatory (mmHg, *X*-axis) techniques in Croatia (*n*=5), group A (yearlings, *n*=6) and group B (subadults and adults, *n*=10) in Scandinavia. Each simultaneous pair of measurements is represented by a fix, with oscillometric and auscultatory values as coordinates (“*y*” and “*x*”, respectively). The identity function (*y*=*x*) corresponds to the maximum concordance between the two techniques.

**Fig. 2:**
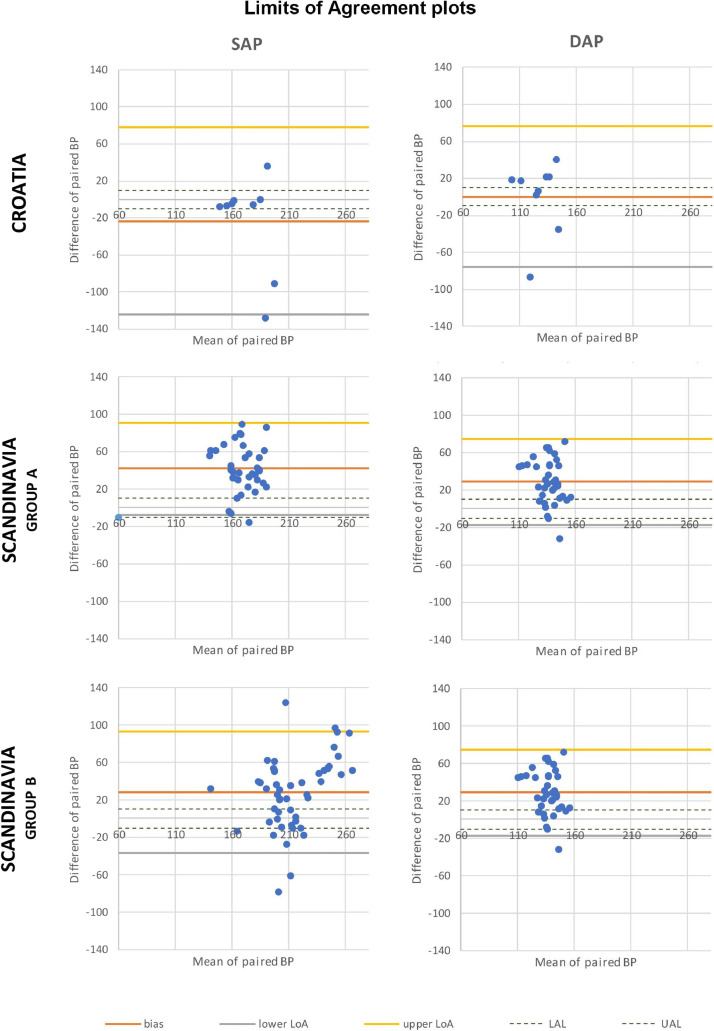
Bland–Altman plots for paired corrected values of SAP and DAP, simultaneously recorded by oscillometric and auscultatory techniques in Croatia (*n*=5), group A (yearlings, *n*=6) and group B (subadults and adults, *n*=10) in Scandinavia. Each fix is obtained by plotting the mean (X-axis) and the difference (*Y*-axis) between paired values. Mean difference (bias), upper LoA (bias + 1.96 SD), and lower LoA (bias – 1.96 SD) between paired values are represented with straight lines. Upper acceptable limit (UAL, 10 mmHg) and lower acceptable limit (LAL, −10 mmHg) are represented with dashed lines.

The correlation between the two methods was poor for SAP and DAP both in Croatia and in Scandinavia, with values of *r* < 0.4 for all groups. The agreement between the two instruments was poor in both studies: 72.2% in Croatia and 56.7% in Scandinavia of the simultaneous concurrent SAP and DAP readings differed by more than 20 mmHg, whereas by more than 50 mmHg in 34.0% and 11.3% of total cases, respectively. More than 95% of the paired values in the Bland-Altman plot lied within the LoAs, nevertheless the resulting agreement interval is too wide to be clinically acceptable (> 90 mmHg in all groups). The auscultatory technique recorded higher values than the oscillometric method for paired SAP, MAP (calculated) and DAP values in 93.9% of total measurements in yearlings, and 75.0% in subadults. On the other hand, this was not always true in adult bears: 75.7% for SAP values, 42.4% for calculated MAP values, and only 24.2% for DAP values resulted higher with the auscultatory technique than with the oscillometric method. Graphs of the Bland-Altman plots and agreement between the two techniques are presented in Figs. 1 and 2.

When looking at the auscultatory BP values from the Croatian sample, either a stable or a slightly decreasing trend over time can be identified for SAP and MAP, while DAP increased over time in one bear. Likewise, a decreasing trend of SAP and MAP (DAP was approximately constant) over time was identified in 17 out of 20 bears in Scandinavia. Furthermore, several BP elevations occurred according to the auscultatory method in 11 out of 20 Scandinavian bears (4 yearlings, 2 subadults and 5 adults). Eight out of those 11 bears were undergoing abdominal surgery when the aforementioned elevations occurred. Although some of the BP variations were consistent between the two techniques, most of the auscultatory trends were in contrast with oscillometric values, which did not follow any constant pattern due to the inconstant reading and to the very high difference among consecutive values. This was true also in Croatia, where neither ketamine administration nor painful stimuli were carried out during the monitoring time of the bears. For these reasons, further data analysis on SAP, MAP (calculated), and DAP values were performed only on measures obtained by the auscultatory technique..

**Fig. 3:**
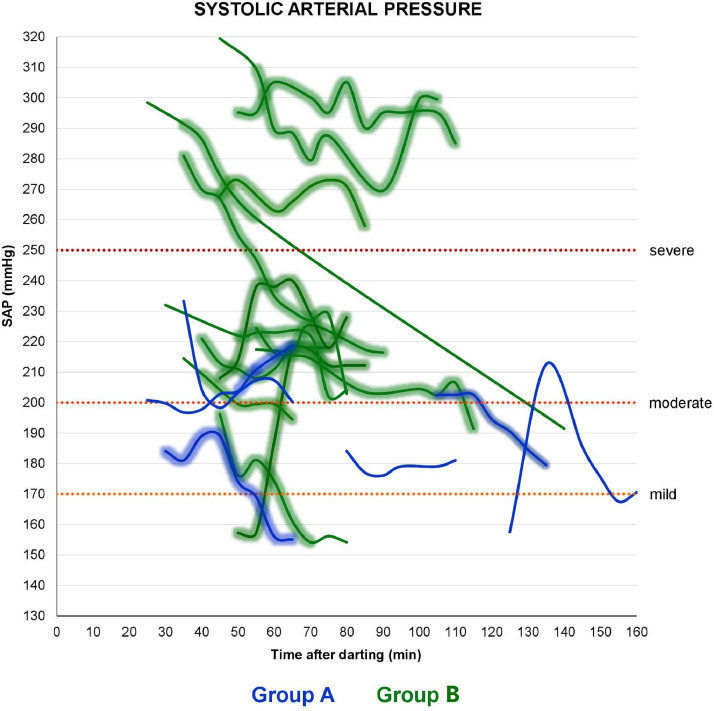
Corrected values of auscultatory SAP in 19 bears captured in Scandinavia during the monitoring time. Each line represents the systolic arterial pressure of one bear, colored in blue for group A (yearlings) and green for group B (subadults and adults). The highlighted segments indicate that the bear is undergoing surgery in that gap of time. In the graph, the mild, moderate and severe systolic hypertension cutoffs are also indicated.

According to the auscultatory method, all bears were hypertensive with varying severity. One bear in Scandinavia developed mild systolic hypertension during the monitoring period, with MAP varying within the reference range. Two adults presented an SAP > 300 mmHg according to the manual sphygmomanometer, though its working range is up to 300 mmHg according to the manufacturer's description (Moretti). Those values were considered as above 300 mmHg during the data analysis. Only one bear restored its blood pressure within the normal range during anesthesia in Scandinavia. SAP was significantly higher in group B than in group A, whereas no significant differences were pointed out for MAP and DAP between groups. HR, SAP, MAP and DAP mean and range values, hypertension levels, and trends of variation of BP in the bears are shown in detail in Tables 2, 3, 4 and Fig. 3, respectively.

**Table 2:** Mean, Standard Deviation (±SD) and range values of heart rate (HR) during the chemical immobilizations in Croatia and within group A (yearlings) and group B (subadults and adults) in Scandinavia.

HR (bpm)	Croatia	Scandinavia	
	/	Group A	Group B
**Mean**	63	86	53
**± SD**	4.47	8.20	4.66
**Range**	40–95	49–129	26–77

**Table 3:** Age, sex, weight, mean ± SD and range values of systolic arterial pressure (SAP), mean arterial pressure (MAP) and diastolic arterial pressure (DAP) for each bear and within groups (TOT.), measured with the Korotkoff's technique throughout 25 chemical immobilizations in Croatia and in Scandinavia. Two bears were excluded (excl.) from the analysis.

CROATIA				SAP Korotkoff (mmHg)			MAP Korotkoff (mmHg)			DAP Korotkoff (mmHg)		
Age		Sex	Weight	Mean	± SD	Range	Mean	± SD	Range	Mean	± SD	Range
6		M	109	185	0.00	185*	144	0.00	144*	123	0.00	123*
1		M	39	163	10.50	153–181	130	12.31	113–143	113	14.36	94–125
8⁎⁎		M⁎⁎	189⁎⁎	171	5.50	162–173	139	6.43	130–146	123	7.37	114–132
8⁎⁎		M⁎⁎	176⁎⁎	243	17.03	218–268	178	13.54	157–193	145	14.83	126–162
9		F	101	excl.	excl.	excl.	excl.	excl.	excl.	excl.	excl.	excl.
TOT.				196	11.04	153–268	151	11.84	113–193	128	11.89	94–162

SCANDINAVIA				SAP Korotkoff (mmHg)			MAP Korotkoff (mmHg)			DAP Korotkoff (mmHg)		
Group	Age	Sex	Weight	Mean	± SD	Range	Mean	± SD	Range	Mean	± SD	Range
A	1	F	18	excl.	excl.	excl.	excl.	excl.	excl.	excl.	excl.	excl.
	1	F	22	179	2.88	176–184	157	3.80	153–163	146	6.34	139–155
	1	F	24	194	9.39	180–203	163	10.83	149–173	147	11.75	133–158
	1	M	21	178	19.18	158–213	155	15.85	138–182	143	14.26	128–167
	1	F	18	175	13.66	155–189	157	11.94	140–171	148	11.26	132–162
	1	F	19	204	7.41	197–219	173	7.35	165–186	158	7.43	150–170
	1	F	18	208	11.76	198–233	180	11.46	165–202	166	11.94	148–187
TOT. A	/			190	10.69	155–233	164	10.20	138–202	152	10.45	128–187
B	16	F	123	169	15.26	154–196	121	3.75	116–127	97	6.36	93–108
	4	F	59	197	26.11	157–217	166	23.41	129–188	148	27.34	105–174
	6	F	83	215	4.59	208–221	178	4.90	173–187	159	7.33	150–170
	6	M	154	219	3.71	216–225	163	4.41	159–170	135	6.40	130–146
	8	M	241	296	5.84	285–305	215	9.70	198–232	174	13.06	155–195
	5	F	61	203	7.53	194–214	160	13.09	146–179	138	15.94	121–161
	3	F	56	227	12.11	208–240	186	5.86	175–192	165	4.02	156–168
	12	F	86	209	9.84	191–224	154	7.44	140–166	127	7.62	114–140
	24	F	90	266	43.89	191–298	195	16.28	170–208	159	5.87	149–164
	8	F	97	268	5.30	258–273	173	4.33	166–180	125	4.92	120–135
	17	F	71	294	15.22	269–319	221	9.61	207–241	184	7.45	176–201
	8	F	87	244	23.98	203–281	163	15.54	133–183	123	11.89	98–134
	12	F	83	218	11.03	202–232	157	9.58	145–170	127	9.76	117–139
TOT. B	/			235	13.69	154–319	175	9.83	116–241	144	10.03	93–201

⁎ Assessed in only one 5-min gap.

⁎⁎ This same bear which was trapped twice in this study.

**Table 4:** Number of bears which reached systolic, diastolic, and overall (based on MAP) hypertension of different seriousness (none, mild, moderate and severe), according to the auscultatory technique, during 23 chemical immobilizations in Croatia (*n* = 4) and Scandinavia (*n* = 19). Differences between group A (yearlings) and group B (subadults and adults) in Scandinavia are pointed out.

	Background	Group	Total	None	Mild	Moderate	Severe
**Systolic hypertension**	**CROATIA**	**/**	4	0	**3**	0	**1**
	**SCANDINAVIA**	**A**	6	0	**2**	**4**	0
		**B**	13	0	**1**	**7**	**5**
**Diastolic hypertension**	**CROATIA**	**/**	4	0	**2**	**2**	0
	**SCANDINAVIA**	**A**	6	0	0	**5**	**1**
		**B**	13	**1**	0	**9**	**3**
Overall hypertension*	**CROATIA**	**/**	4	0	**3**	**1**	0
	**SCANDINAVIA**	**A**	6	0	0	**5**	1
		**B**	13	**1**	0	**9**	**3**
**Hypertension at the end of monitoring**⁎⁎	**CROATIA**	**/**	4	**1**	**2**	**1**	0
	**SCANDINAVIA**	**A**	6	0	**3**	**3**	0
		**B**	13	**1**	**4**	**6**	**2**

⁎ It refers to MAP increases.

⁎⁎ It is based on the last MAP recorded value at the end of the monitoring time, just before anesthetic reversal.

The modeled data set contained 19 bears and 128 observations. The selected top model contained the time, age and surgery state as significant variables. The smoother on time used 1 degree of freedom, indicating a linear decrease and the smoother was therefore removed. MAP decreased by approximately one unit every 3 minutes and age increased MAP by 1.65 units per year. During surgery, MAP decreased by 6.71 units (Table 5). Data of one bear was removed because the MAP measurements were done considerably later than in all the other bears and one observation was removed because of a >80 min time gap to the prior one.

**Table 5:** Summary statistics of the selected top model. Estimates are in MAP units. The S(ID, Time, “re”) is fitted as factor smooth interaction between the individual bear over time and represents the random component of the model. The correlation parameter (*ρ*) is used to fit an AR1 structure.

	Estimate	Standard error	*T*-value	*P*-value
Intercept	175.39	5.79	30.29	<0.001
Time	−0.29	0.10	−2.99	0.003
Age	1.65	0.50	3.28	0.001
Surgery status On	−6.71	2.59	−2.596	0.011

	Estimated df	Reference df	*F*-value	*P*-value
S(ID, Time, “re”)	15.37	17	19.69	<0.001
*Ρ*	0.6			
Adjusted *R*^2^	0.78			

## Discussion

The present study showed that the auscultatory technique can be reliably used in brown bears to monitor BP in field conditions. The measurements obtained confirm that the capture methods combined with the drugs used in the study, generally produce transient hypertension in bears, with a significantly higher severity in adults and subadults.

The duration of exertion was considerably longer in Croatia than in Scandinavia, in some cases more than 50 times longer, due to the long distance from the capture team to the trap sites. Nevertheless, since the type of exertion was different, the resulting impact on physiology was also different.

Both drug combinations provided a fast and predictable induction of anesthesia, with a shorter mean onset time for MZT, however, in both contexts, additional ketamine administration was sometimes required to prolong the anesthesia. Immobilization with XK elicited an overall good muscular relaxation and sedation, but resulted in more difficult assessment of the anesthetic depth, thus requiring higher expertise and attention from the team.

It has been well documented that α_2_-agonist administration results in bradycardia, partially secondary to the induced systemic hypertension, especially in the early phases after initial administration and tends to decrease over time. In contrast, we observed an overall decreasing trend of HR in both capture contexts (21 of 25 bears) extending up to two hours after darting, as similar studies previously reported (Caulkett & Cattet, 1997; Caulkett et al., 1999; Lima et al., 2016; Selmi et al., 2004), and was more marked in yearlings. This might be due to a prolonged decrease in HR as it happens in humans (Murrell & Hellebrekers, 2005) or to the combination of the hemodynamic effects of the administered drugs: the initial tachycardia elicited by both the exertion (trapping or chase) and the dissociative anesthetic (ketamine or tiletamine) is probably overcome by the prolonged α_2_-induced bradycardia, as high doses are documented to prolong the duration of sedative and hemodynamic effects (Kuusela et al., 2000). Regarding the age groups, HR was significantly higher in group A than in group B (up to 129 bpm vs. 77 bpm), with females presenting the highest rates in both groups, as previously reported in other studies (Fahlman et al., 2011). Additionally, two bears from the Scandinavian sample developed marked bradycardia with HR < 35 bpm.

### Correlation and agreement between the two monitoring methods

In this study, Korotkoff's auscultatory technique was adopted for non-invasive blood pressure monitoring in free-ranging brown bears. This method is not usually reliable in small animal veterinary practice, due to the difficulty in placing both the cuff and the stethoscope's bell over a sufficiently long, straight and superficial artery. Nevertheless, it is routinely adopted in human medical clinic settings (Carlson et al., 2019) and sometimes preferred to standard oscillometry (Canzanello et al., 2001).

Korotkoff's auscultatory technique proved to be markedly more successful, more precise, and overall more reliable for BP monitoring than the oscillometric method in field circumstances in brown bears. However, a quiet environment and shaving the limb were essential for improving the quality of auscultation. Korotkoff's technique provided clear auscultation in 93.3% of total attempts, thus allowing to easily determine SAP and DAP values without needing to repeat the measurement, compared to only the 29.3% (with apparently higher success rate when the cuff was placed around the forearm) with the automatic oscillometer. Shaved and perfectly immobile limbs were essential to make the oscillometric device work, making it time-consuming and unreliable in some field circumstances. In three Scandinavian bears, it could not measure any value throughout the whole anesthesia, even after shaving the limb, pouring the soap solution and replacing the cuff. In one of those bears, whose SAP was above 300 mmHg according to the standard manual sphygmomanometer, the automatic device aborted the measurement probably because it was beyond the range of values it could detect. Nevertheless, in one yearling, the techniques showed very high agreement and repeatability among values, forming two trends of variations, consistent with each other, throughout all the monitoring period.

This unpredictably low efficacy could be explained by either an inappropriate algorithm of the automatic sphygmomanometer model, an occasional occurrence of bradyarrhythmias (as commonly reported after α_2_-agonist administration) (Bordley et al., 1951), or marked vasoconstriction. Inappropriate cuff placement is not likely to contribute to the failure of the readings because all cuff sites were accurately shaved, wet and the cuff was placed following the most recent AHA reference guidelines (Whelton et al., 2017). Further, low ambient temperatures, with 4°C the lowest, would presumably not had a relevant negative impact on the device functionality, as no association between measurement failure and ambient temperature or monitoring time (or day) was demonstrated. Eventually, all test measurements on the operator were successful and concordant with the auscultatory technique.

The poor correlation and agreement between the two techniques is in contrast to previous findings in comparative studies between oscillometry and invasive BP monitoring in both captive and wild anesthetized brown bears, which resulted in a good degree of accuracy (Teisberg et al., 2014). The poor correlation in the present study is probably linked to the same causes of the low efficacy of the automatic oscillometer.

Although we have no documentation of its accuracy in brown bears, the auscultatory technique detected generally higher SAP values, compared to oscillometry, in Croatia, thus detecting severe systolic hypertension in one bear, which was not diagnosed with oscillometry. Likewise, the manual sphygmomanometer detected higher values compared to the automatic instrument in both groups in Scandinavia and for both SAP and DAP, although in a lower percentage of cases and to a less marked extent.

Former studies in men and children have found BP obtained using oscillometric monitors to be either higher (Carlson et al., 2019; Eliasdottir et al., 2013; Wong et al., 2001) or lower (Wong et al., 2001) than with manual sphygmomanometers, likely due to the different automated oscillometers used in each study. The fact that DAP seems to be lower with the oscillometric method may either suggest difficulties in the determination of the fifth Korotkoff sound with the auscultatory technique or the fact that the oscillometric monitor detects no actual DAP, but vibrations in the arterial wall (Eliasdottir et al., 2013).

### Hypertension in Croatia and Scandinavia

Hypertension of different severity was identified by the auscultatory method in all bears in both capture settings, and this was consistent with intra-arterial values presented in previous studies carried out on bears immobilized by using these drug combinations and capture methods (Caulkett & Cattet, 1997; Teisberg et al., 2014). In the present study BP significantly decreased over time, especially SAP, but did not reach the normotensive range in 21 of 23 bears. In fact, average SAP kept decreasing until the end of BP monitoring in Scandinavia, without stabilizing. This is most likely due to the long half-life of the drug combination when administered at elevated doses (Kuusela et al., 2000). Considering the above-mentioned points and since none of the sedative and anesthetic drugs present in the darts are known to elicit relevant hypotensive adverse effects on bears, it can be assumed that the proposed baseline ranges for SAP/DAP of 110–170/70–110 mmHg (Siegal-Willott, unpublished data 2018, based on 29 indirect BP measurements on unanesthetized trained bears at Smithsonian's National Zoo) are either reasonable or just slightly lower than the true baseline BP.

Korotkoff's technique pointed out a more marked hypertension in the Scandinavian sample, especially regarding SAP in group B, with a relevant time-dependent drop.

It is well documented that systolic hypertension is generally more harmful than diastolic hypertension in the short-term (Brown et al., 2007; Malmstein, 2007). Although the ranges of SAP values were not relevantly different between the two scenarios (153–268 mmHg in Croatia and 154–319 mmHg in Scandinavia), 3 of 4 bears in Croatia achieved at most a mild systolic hypertension, whereas 84.2% of bears developed moderate to severe systolic hypertension in Scandinavia. Furthermore, two adult bears chemically immobilized in Scandinavia maintained severe hypertension for at least 110 min before the administration of atipamezole, with SAP and MAP values ranging between 269–319 mmHg and 198–241 mmHg, respectively. Additionally, it is documented that the auscultatory method underestimates SAP, when compared with gold standard techniques (Avolio et al., 2010; Bordley et al., 1951; Carlson et al., 2019; Ghione et al., 1998; Hunyor et al., 1978; Marks & Groch, 2000), therefore we could expect even higher SAP values in our case. High DAP is also dangerous both during sudden BP rises and when chronically established. While only mild and moderate diastolic hypertension occurred in Croatia, higher values were achieved in Scandinavia by both groups, except from one bear that did not develop diastolic hypertension. These relevant differences could be explained either by the higher vasoconstrictive effects of the medetomidine administered doses compared to xylazine (Sinclair, 2003), or by the possibly greater acute hemodynamic impact of the chase compared to the snare, or by the fact that all Scandinavian bears underwent abdominal surgery which may have led to nociception and consequent rises of BP, or by intrinsic differences between bear samples, or all of the above.

The BP results of group B are consistent with other studies on wild grizzly bears darted from the helicopter with dexmedetomidine-zolazepam-tiletamine (Teisberg et al., 2014) and on wild black bears immobilized with MZT in culvert traps (Caulkett & Cattet, 1997). Nevertheless, mean MAP values of group B in the current study were slightly lower than the ones documented in trapped adult polar bears (Caulkett et al., 1999) darted with similar MZT doses (mean direct MAP of 219 ± 22 mmHg), which are more similar to the severely hypertensive bears identified in our study.

### Impact factors within the Scandinavian capture context

The model selection indicated that the age of the captured bear significantly influenced BP, consistent with studies in dogs (Bodey & Michell, 1996) whose BP increases by 1–3 mmHg/year of age. This is possibly due to higher pulse pressure and impaired renal function, which are common findings in older individuals. The slight decrease of BP related to the abdominal surgery is more controversial. This can be explained by the dorsal recumbency of the bears during surgery, so impaired venous return to the heart due to the compression of large veins elicited by the weight of the abdominal organs. This is a common finding when changing recumbency, however surgery itself may sometimes be responsible for transient BP increases. An overall good degree of somatic analgesia is consistent with MZT administration at similar doses in bear species (Caulkett et al., 1999), and in several other studies in veterinary literature (Murrell & Hellebrekers, 2005). Nevertheless, visceral analgesia may be inadequate, particularly in the later phases of the surgery and in case of marked nociceptive stimulation. Although these stimuli are not perceived as pain, nociception reveals itself with HR and BP increments (Caulkett et al., 1999; Haga et al., 2001). This likely could explain the peaks in SAP and DAP during the abdominal surgery in some bears, despite the mild drop of BP pointed out by the statistical model compared to pre-surgery levels.

### Limits of the research

The overall small number of bears and the difference in size between the Croatian and the Scandinavian sample prevented us from carrying out some relevant inferences and comparisons between the two immobilization contexts. Besides, since different brands of oscillometric recorders use different algorithms, we are aware of the fact that results from one study cannot be inferred for all oscillometric automated devices (Chiolero et al., 2014; Ogedegbe & Pickering, 2010). The corrective calculation based on the optimal CW/LC of 0.41 that we carried out on BP values, assumes a fixed optimal ratio for both SAP and DAP values and all arm circumferences of the study. This is a one-century-old very argued issue in human medicine, as some previous studies confirmed that DAP is systematically overestimated with such CW/LC and that as arm circumference increases, the actual optimum ratio decreases (Marks & Groch, 2000). No similar studies have been carried out on bears, so we used the most recent guidelines for bear anesthesia as references (Teisberg et al., 2014; West et al., 2018). Further studies with a more significant number of subjects, and gold standard techniques used as validation, would better assess the effects on hemodynamics elicited by the sum of the single factors in Croatia and Scandinavia. Additionally, the baseline BP reference issue in brown bears remains still unsolved as well as the degree of hypertension that could be considered either physiological or harmless, since no follow-up studies dealing with the incidence of immobilization-induced target-organ damage have been carried out so far.

## Conclusions

Korotkoff's technique for non-invasive BP monitoring has been successfully performed on free-ranging brown bears, providing evidence of overall good repeatability and feasibility to be carried out in field conditions. On the contrary, the automated oscillometer failed in assessing BP in most cases in this study, and the recorded values did not correlate with the ones gained by the manual aneroid sphygmomanometer, neither in Croatia nor in Scandinavia. Simultaneous intra-arterial BP monitoring would be essential to assess the accuracy of the auscultatory technique as a quick, easy, non-invasive BP measurement method during brown bear chemical immobilization in the field.

The occurrence of different degrees of hypertension was confirmed in all the bears of the study, with higher values in Scandinavia than in Croatia, and with significantly higher systolic values in subadults and adults than in yearlings. This was associated with significantly higher HR in yearlings. As both the trends of variation of BP, especially for SAP, and the statistic model confirmed, BP was significantly decreasing over time in most of the bears, it was increased in older individuals, and the concurrent visceral surgery was associated with slightly lower MAP values and occasional transient increases of SAP.

## Funding

The Croatian part of the study was supported by the Faculty of Veterinary Medicine, University of Zagreb, Croatia within the national project “Study of numbers, space use and behaviour of bears in the area of Plitvice Lakes National Park” (*Istraživanje brojnosti, korištenja prostora i ponašanja medvjeda na području NP Plitvička jezera*) and by EURONATUR and BERND THIES foundation. The Scandinavian part was supported by the Inland Norway University of Applied Sciences, Norway, and the Scandinavian Brown Bear Research Project (*Skandinaviska Björnprojektet*). Financial support was provided to JM also by the European Commission under the Erasmus+ program. The purchase of both manual and automatic sphygmomanometers was carried out by AB.

## Ethical permits

Croatian responsible ministries approved the part of the research in Croatia, Ministry of Environment and Nature Protection (KLASA: UP/I-612-07/15-48/47, URBROJ: 517-07-1-1-1-15-4, Zagreb, Croatia) and Ministry of Agriculture (KLASA: UP/I-323-03/16-01/123, URBROJ: 525-11/1029-16-2, Zagreb, Croatia). The experiments in Sweden were approved by the Swedish Ethical Committee on Animal Research (Uppsala, Sweden; #C18/15), the Swedish Environmental Protection Agency (Stockholm, Sweden; NV-0758-14), and the Swedish Board of Agriculture (#31-11102/12).

## Declaration of Competing Interest

The authors declare that the research was conducted in the absence of any commercial or financial relationships that could be construed as a potential conflict of interest.
